# Correction to: Altered microstructure of the contralesional ventral premotor cortex and motor output after stroke

**DOI:** 10.1093/braincomms/fcad248

**Published:** 2023-09-29

This is a correction to: Paweł P Wróbel, Stephanie Guder, Jan F Feldheim, José A Graterol Pérez, Benedikt M Frey, Chi-un Choe, Marlene Bönstrup, Bastian Cheng, Yogesh Rathi, Ofer Pasternak, Götz Thomalla, Christian Gerloff, Martha E Shenton, Robert Schulz, Altered microstructure of the contralesional ventral premotor cortex and motor output after stroke, *Brain Communications*, Volume 5, Issue 3, 2023, fcad160, https://doi.org/10.1093/braincomms/fcad160

The Funding section of the originally published version of this manuscript has been corrected.

